# Quercetin Improves Mitochondrial Function and Inflammation in H_2_O_2_-Induced Oxidative Stress Damage in the Gastric Mucosal Epithelial Cell by Regulating the PI3K/AKT Signaling Pathway

**DOI:** 10.1155/2021/1386078

**Published:** 2021-11-27

**Authors:** Xueting Yao, Yingbing Mei, Wanyu Mao

**Affiliations:** ^1^Department of Traditional Chinese Medicine, Wuhan Third Hospital, Wuhan, China; ^2^Department of Geriatrics, Hubei Provincial Hospital of Traditional Chinese Medicine, Hubei, China; ^3^2019 Master's Degree Candidate, Hubei University of Chinese Medicine, Hubei, China

## Abstract

Functional dyspepsia (FD) is one of the most common functional gastrointestinal disorders, the therapeutic strategy of which it is limited due to its complex pathogenesis. Oxidative stress-induced damage in gastric mucosal epithelial cells is related to the pathogenesis and development of FD. Quercetin (Que) is one of the active ingredients of Zhishi that showed antioxidant, antiapoptotic, and anti-inflammatory effects. The aim of this study is to investigate the effect of Que on oxidative stress-induced gastric mucosal epithelial cells damage and its underlying molecular mechanism. The gastric mucosal epithelial cell line GES-1 was treated with 200 *μ*M of H_2_O_2_ to construct an oxidative stress-induced damage model. The H_2_O_2_ cells were then administrated with different concentrations of Que. The results indicated that high concentration of Que (100 *μ*M) showed cytotoxicity in H_2_O_2_-induced GES-1 cells. However, appropriate concentration of Que (25 and 50 *μ*M) alleviated the oxidative stress damage induced by H_2_O_2_, as demonstrated by the increase of proliferation, decrease of ROS generation, apoptosis, inflammation, and alleviation of mitochondrial function and cell barrier. In addition, Que increased the activation of phosphorylation of PI3K and AKT decreased by H_2_O_2_. To investigate whether Que alleviated the oxidative stress damage in GES-1 cells by the PI3K/AKT signaling pathway, the GES-1 cells were treated with Que (25 *μ*M) combined with and without LY294002, the PI3K inhibitor. The results showed that LY294002 suppressed the alleviation effect on Que in H_2_O_2_-induced GES-1 cells. In conclusion, the current study demonstrates that Que alleviates oxidative stress damage in GES-1 cells by improving mitochondrial function and mucosal barrier and suppressing inflammation through regulating the PI3K/AKT signaling pathway, indicating the potential therapeutic effects of Que on FD.

## 1. Introduction

Functional dyspepsia (FD) is one of the most common functional gastrointestinal disorders, comprising three subtypes based on pathophysiology and aetiology: epigastric pain syndrome, postprandial distress syndrome, and a subtype with overlapping epigastric pain syndrome and postprandial distress syndrome [1]. The prevalence of FD varies widely worldwide. According to their respective definitions of FD, it is generally as high as 10–40% in Western countries and range from 5% to 30% in Asia [2]. Presently, the drug treatment for FD mainly focused on its subtypes, the use of motility agents and gastric basal relaxants to relieve postprandial distress syndrome and the use of acid inhibitors to treat epigastric pain syndrome. In addition, central active nerve modulators and Chinese herbs also play a role in the treatment of FD [1]. However, the therapy of FD is limited due to its complex pathogenesis. Therefore, understanding the pathogenesis of FD and finding the appropriate treatment drugs are the focus of current research on FD.

Oxidative stress refers to the physiological and pathological reactions of cells and tissues caused by excessive production of high active molecules such as reactive oxygen species (ROS) and imbalance of oxidation and antioxidation in the body when the body is exposed to various harmful stimuli. ROS is mainly generated from mitochondria. Excess production of ROS induces the decrease of mitochondrial membrane potential by changing the permeability of mitochondrial membrane, leading to a series of caspases-related cascade reactions, thereby promoting mitochondrial apoptosis. Mitochondria are important organelles with a double membrane structure and are the most important site of ATP production, playing an important role in the regulation of oxidative stress, calcium homeostasis, and apoptosis in cells [3]. Its morphological changes are regulated by mitochondrial fusion and fission, and its kinetic disturbance can lead to excessive ROS production and apoptosis [4,5]. More ROS will be produced after mitochondrial injury, which accelerates cell apoptosis [6]. Evidence demonstrated that oxidative stress-induced damage in gastric mucosal epithelial cells is related to the pathogenesis and development of FD [7]. Antioxidant agent mitoTEMPO showed gastroprotection by preventing mitochondrial apoptosis and mitochondrial oxidative stress-mediated proinflammation [8]. Collectively, reducing the level of oxidative stress may alleviate FD.

Evidence demonstrated that a variety of traditional Chinese medicines show great therapeutic effect on FD [9–11]. Zhishi is the immature fruit of *Citrus sinensis Osbeck* or *Citrus aurantium* L. that showed excellent therapeutic effect on abdominal distension, abdominal pain, diarrhea, constipation, and slow gastrointestinal peristalsis caused by FD [12]. Our previous study found that Zhishi promoted the differentiation and proliferation of gastric interstitial cells of Cajal in functional dyspepsia rats and improved the activity of damaged interstitial cells of Cajal, thereby regulating gastrointestinal motility, indicating the improvement effect of Zhishi on gastric motility [13,14]. In addition, network pharmacology analyzed predicted the treatment effect of Zhishi on FD. GO enrichment analysis showed that the active ingredients in Zhishi are closely related to FD, and pathway analysis revealed that Zhishi exerts synergistic effects on FD treatment by acting on genes upstream and downstream of the calcium signaling pathway, cAMP signaling pathway, MAPK signaling pathway, and NF-*κ*B pathway [15]. However, the specific molecular mechanism needs to be clarified. Quercetin (Que) is one of an active ingredients of Zhishi [16] that showed antioxidant, antiapoptotic, antithrombotic, and anti-inflammatory effects by regulating various signaling pathways, especially PI3K/Akt/mTOR signaling pathway [17,18]. Studies have reported that Que effectively inhibited the damage of gastric mucosa epithelial cells caused by oxidative stress and thus protected gastric mucosa from stimulation by various factors [19,20]. However, whether Que has therapeutic effect on FD by repairing oxidative stress-induced gastric mucosal epithelial cells damage has been rarely reported.

The aim of this study is to investigate the effect of Que on oxidative stress-induced gastric mucosal epithelial cells damage and its underlying molecular mechanism. Herein, the gastric mucosal epithelial cells were treated with 200 *μ*M H_2_O_2_ to construct an oxidative stress-induced damage model. The H_2_O_2_ cells were then administrated with Que to evaluate its effect on oxidative stress-induced damage in gastric mucosal epithelial cells.

## 2. Materials and Methods

### 2.1. Cell Culture and Treatment

The human gastric mucosal epithelial cell line GES-1, supplied by iCell, was cultured in Dulbecco's modified eagle medium (Hyclon) supplemented with 10% fetal bovine serum (FBS, Gibco) and maintained at 37°C with 5% CO_2_. 100 mg of quercetin (IQ0010, Solarbio) dissolved in 2 ml of DMSO, the molar concentration of the mother liquor is 165.43 mM, and different concentrations of quercetin are prepared according to the steps in the product specification. When the confluence reached 80–90%, the cells were treated for 24 h with different concentration of quercetin (Que, 0, 12.5, 25, 50, and 100 *μ*M), followed by addition 200 *μ*M H_2_O_2_ treatment. The cells in control group were treated with normal medium. The cells in the model group were only treated with 200 *μ*M H_2_O_2_ [20]. The cell proliferation, apoptosis, inflammation, mitochondrial function, barrier, and the activity of PI3K/AKT pathway were then evaluated. Subsequently, GES-1 cells were treated with 10 *μ*M LY294002, the inhibition of PI3K [21,22], combination with/without 25 *μ*M Que, followed by treating with 200 *μ*M H_2_O_2_. The cell apoptosis, mitochondrial function, and the activity of PI3K/AKT pathway were then evaluated.

### 2.2. Cell Counting Kit-8 Assay

The harvested cells were seeded into the 96-well plate at the density of 3 × 10^3^ cells per well and maintained at 37°C with 5% CO_2_ overnight. When the confluence reached 80–90%, the cells were treated for 24 h with different concentration of quercetin (Que, 0, 12.5, 25, 50, and 100 *μ*M) [20], followed by addition 200 *μ*M H_2_O_2_ treatment. The cells were then treated with addition of 10 *μ*l of cell counting kit-8 (CCK-8) solution (Solarbio) and cultured for 4 h. Then, the cells were subjected to a microplate reader (Allsheng) to detect the optical density of cell at 450 nm.

### 2.3. ROS Generation Assay

The harvested cells at the density of 3 × 10^3^ cells/ml were resuspended in 1 ml of diluted DCFH-DA (10 *μ*mol/l) and cultured for 20 min at 37°C with 5% CO_2_. Thereafter, the cells were resuspended in 500 *μ*l of phosphate-buffered saline (PBS) and subjected to the flow cytometry (ACEA Biosciences) to detect the proportion of cells with increase in ROS.

### 2.4. Apoptosis Assay

The AnnexinV-fluorescein isothiocyanate (FITC)/prodium iodide (PI) apoptosis detection kit (BD) was used to evaluate the apoptosis of GES-1 cells. 1 × 10^6^ GES-1 cells were centrifuged for 5 min at 400 × g, 4°C and resuspended in 200 *μ*l of phosphate-buffered saline (PBS). The cells were then stained for 30 min with 10 *μ*l of Annexin V-FITC and 10 *μ*l of PI in the dark. After 300 *μ*l of PBS adding, the cells were subjected to flow cytometry (ACEA Biosciences) to detect the apoptosis rate.

### 2.5. The mRNA Expression of Interleukin-10 and Tumor Necrosis Factor-*α* Assay

Quantitative reverse transcription polymerase chain reaction (qRT-PCR) was performed to detect the mRNA expression of interleukin-10 (IL-10) and tumor necrosis factor-*α* (TNF-*α*) in GES-1 cells. Total RNA was extracted from GES-1 cells using Trizol (Ambion) and reversed-transfected into cDNA. The cDNA were then amplified using PCR. The primary sequences were as follows: IL-10, forward, 5′- ACCAAGACCCAGACAT -3′, reverse, 5′- TCACAGGGAAGAAATC -3'; tumour necrosis factor alpha (TNF-*α*), forward, 5′- CAGGCGGTGCTTGTTC-3′, reverse, 5′- TGTCACTCGGGGTTCG-3'; GAPDH (housekeeping), forward, 5′- GGGAAACTGTGGCGTGAT-3′, reverse, 5′- GAGTGGGTGTCGCTGTTGA-3'. Relative mRNA expression levels were calculated using the 2^-△△Ct^ method.

### 2.6. Mitochondrial Membrane Potential Assay

Flow cytometry was performed to detect the mitochondrial membrane potential (MMP) in GES-1 cells. The harvested GES-1 cells at the density of 1 × 10^6^ in each group were resuspended in 0.5 ml of medium and 0.5 ml of JC-1 staining solution (Beyotime) and cultured for 20 min at 37°C with 5% CO_2_. Thereafter, the cells were centrifuged for 3 min at 400 × g, 4°C and resuspended in 1 ml of JC-1 staining solution and centrifuged for 3 min at 400 × g, 4°C. The cells were then resuspended in 400 *μ*l of JC-1 staining solution and subjected to flow cytometry (ACEA Biosciences) to detect the proportion of cells with decrease in MMP.

### 2.7. Ultrastructure of Mitochondria Observation

Transmission electron microscopy (TEM) was performed to observe the ultrastructure of mitochondria in GES-1 cells. 1 × 107 cells in each group were fixed for 30 min in 2 ml 2.5% glutaraldehyde at 4°C and then fixed for 1 h in 1% osmic acid. After dehydrating, soaking, and embedding, ultrathin sections (∼60 nm) were obtained and stained with uranyl acetate for 20 min and lead citrate for 15 min in the dark. The ultrastructure of mitochondria was observed under the TEM (Hitachi).

### 2.8. Western Blot Assay

Total proteins were extracted from GES-1 cells using radioimmunoprecipitation assay lysis buffer (Solarbio), and protein concentration was quantified using a bicinchoninic acid assay kit (Solarbio). Proteins were separated by sodium dodecyl sulfate-polyacrylamide gel electrophoresis and transferred onto polyvinylidene fluoride membranes. After blocking with 5% skim milk, the membranes were cultured for 1 h with primary antibodies against BCL-2 associated *X* protein (BaX) (1 : 1000, Bioswamp, PAB30040), B-cell lymphoma-2 (Bcl-2) (1 : 1000, Bioswamp, PAB33482), cleaved caspase 3 (1 : 1000, Abcam, Ab2302), density-regulated protein 1 (DRP1) (1 : 1000, Bioswamp, PAB33409) phosphorylation (p)-DRP1 (1 : 1000, Abcam, ab193216), mitochondrial profusion protein Mitofusins (Mfn2) (1 : 1000, Bioswamp, PAB41825), occludin (1 : 1000, Bioswamp, PAB33418), claudin-1 (1 : 1000, Bioswamp, PAB33267), zona occludens 1(ZO-1) (1 : 1000, Bioswamp, PAB36669), phosphatidylinositol-3 kinase (PI3K) (1 : 1000, Bioswamp, PAB30084), p-PI3K (1 : 1000, Abcam, Ab182651), AKT (1 : 1000, Bioswamp, PAB34089), p-AKT (1 : 1000, Abcam, Ab38449), and GAPDH (1 : 1000, Housekeeping, Bioswamp, PAB36269), followed by 1 h of incubation with goat anti-rabbit IgG secondary antibody (1 : 20000, Bioswamp, SAB43714).

### 2.9. Statistical Analysis

Data are represented as the mean ± standard deviation (SD). Differences among groups were analyzed using one-way analysis of variance followed by Tukey's test. *P* < 0.05 was considered to be statistically significant.

## 3. Results

Que alleviates H_2_O_2_-induced oxidative stress, apoptosis, and inflammatory in GES-1 cells.

**Figure 1:**
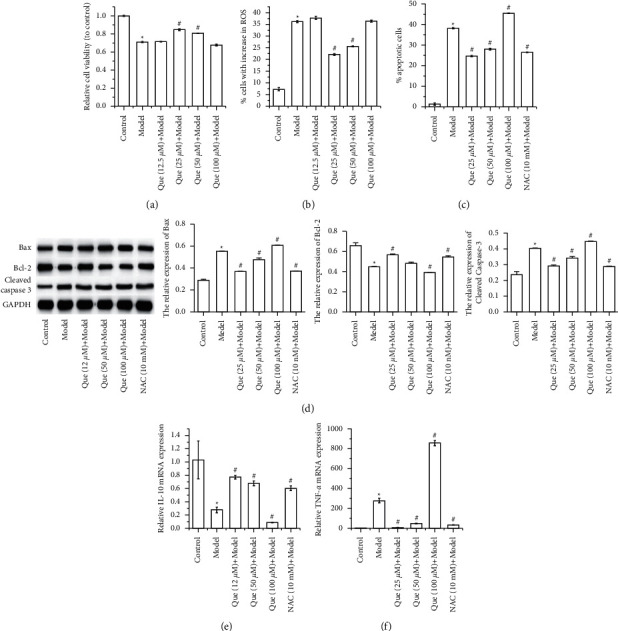
Que alleviates H_2_O_2_-induced oxidative stress, apoptosis, and inflammatory in GES-1 cells. (a) CCK-8 was performed to measure the viability of GES-1 cells. Flow cytometry was performed to evaluate the proportion of (b) cells with increase in ROS and (c) apoptotic cells. (d) Western blot was performed to detect the apoptosis-related proteins Bax, Bcl-2, and cleaved caspase 3 expression in GES-1 cells. qRT-PCR was performed to detect mRNA expression of (e) IL-10 and (f) TNF-*α* in GES-1 cells. Data presets as mean ± SD, *n*− = 3, ^∗^*P* < 0.05 vs. control group, ^#^*P* < 0.05 vs. model group.

As shown in Figures 1(a) and 1(b), 200 *μ*l of H_2_O_2_ decreased the viability and increased ROS generation in GES-1 cells. Low concentration of Que (12.5 *μ*M) showed no effect on H_2_O_2_-induced damage in GES-1 cells, whereas 25 *μ*M of Que alleviated H_2_O_2_-induced damage, as indicated by the increase of viability and the decrease of ROS generation in GES-1 cells compared to the model group. The results also showed that high concentration of Que (100 *μ*M) demonstrated cytotoxicity in GES-1 cells. To further investigate the antioxidant of Que in GES-1 cells, the NAC, an ROS inhibitor, was used as a negative control. Flow cytometry showed that H_2_O_2_-induced apoptosis in GES-1 cells was suppressed by 25 *μ*M and 50 *μ*M of Que and further increased by a high concentration of Que (100 *μ*M) (Figure 1(c)). Western blot assay suggested that 25 *μ*M and 50 *μ*M of Que decreased the activation of Bax and cleaved caspase 3 increased by H_2_O_2_ and increased the activation of Bcl-2 decreased by H_2_O_2_ in GES-1 cells (Figure 1(d)). In addition, H_2_O_2_ induced inflammatory response in GES-1 cells by suppressed the level of IL-10 and enhanced the level of TNF-*α*. The inflammatory response induced by H_2_O_2_ in GES-1 cells was improved by 25 *μ*M and 50 *μ*M of Que (Figures 1(e) and 1(f)).

The effect of 25 *μ*M and 50 *μ*M of Que in H_2_O_2_-induced GES-1 cells was similar to that of NAC.

### 3.1. Que Alleviates H_2_O_2_-Induced Mitochondria Dysfunction in GES-1 Cells

**Figure 2:**
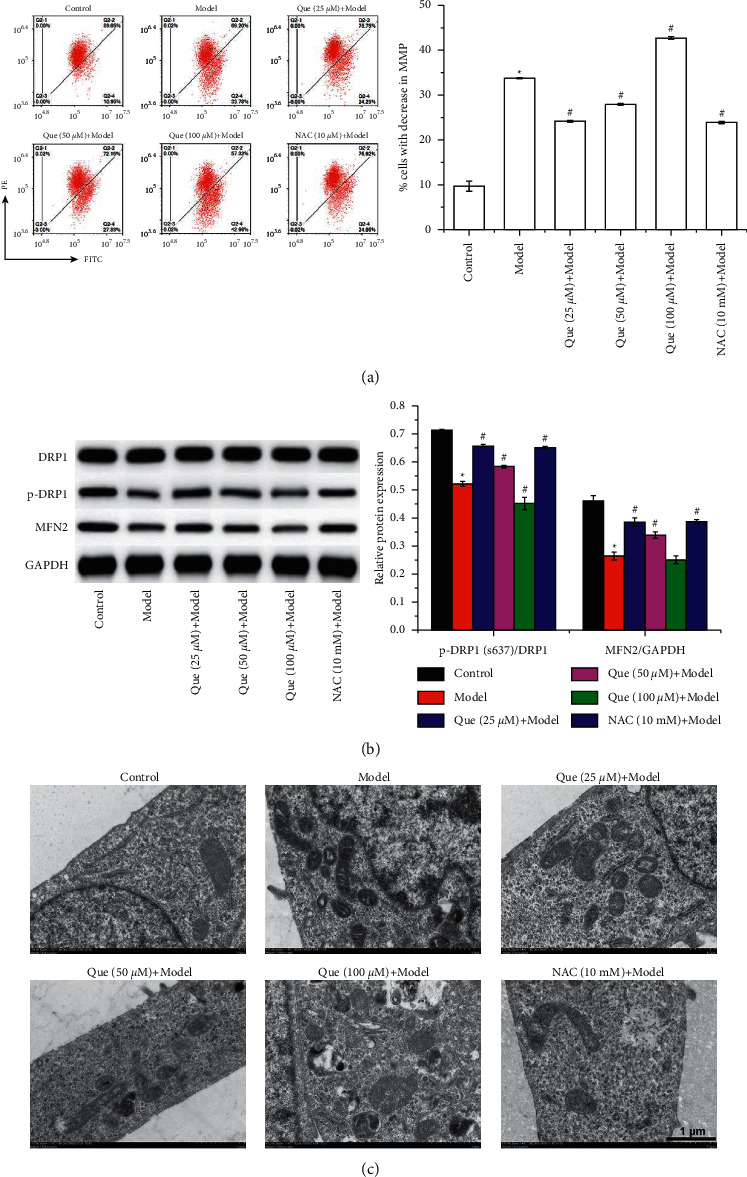
Que alleviates H_2_O_2_-induced mitochondria dysfunction in GES-1 cells. (a) Flow cytometry was performed to measure the proportion of cells with decrease in MMP. (b) Western blot was performed to evaluate the phosphorylation of DRP1 and the expression of DRP1 and MFN2 in GES-1 cells. (c) The representative ultrastructure of mitochondria in GES-1 cells (12.0 K). Data presets as mean ± SD, n− = 3, ^∗^*P* < 0.05 vs. control group, ^#^*P* < 0.05 vs. model group.

Flow cytometry demonstrated that H_2_O_2_ treatment increased the proportion of GES-1 cells with an increase in mitochondrial membrane potential (MMP), which was decreased by 25 *μ*M and 50 *μ*M of Que treatment and further increased by 100 *μ*M of Que treatment (Figure 2(a)). Western blot assay showed that H_2_O_2_ treatment decreased the phosphorylation of DRP1 and the expression of MFN2, which were increased by 25 *μ*M and 50 *μ*M of Que treatment (Figure 2(b)). The effect of NAC on MMP and p-DRP1 and MFN2 expression in GES-1 cells was similar to that of 25 *μ*M and 50 *μ*M of Que. In addition, H_2_O_2_ treatment destroyed the mitochondrial structure in GES-1 cells, as indicated by the swollen of mitochondria and malformation of ridges. The destroyed mitochondrial structure was improved by 25 *μ*M and 50 *μ*M of Que and NAC treatment (Figure 2(c)).

Que improves the cell barrier and activates the PI3K/AKT signaling pathway in H2O2-induced GES-1 cells.

**Figure 3:**
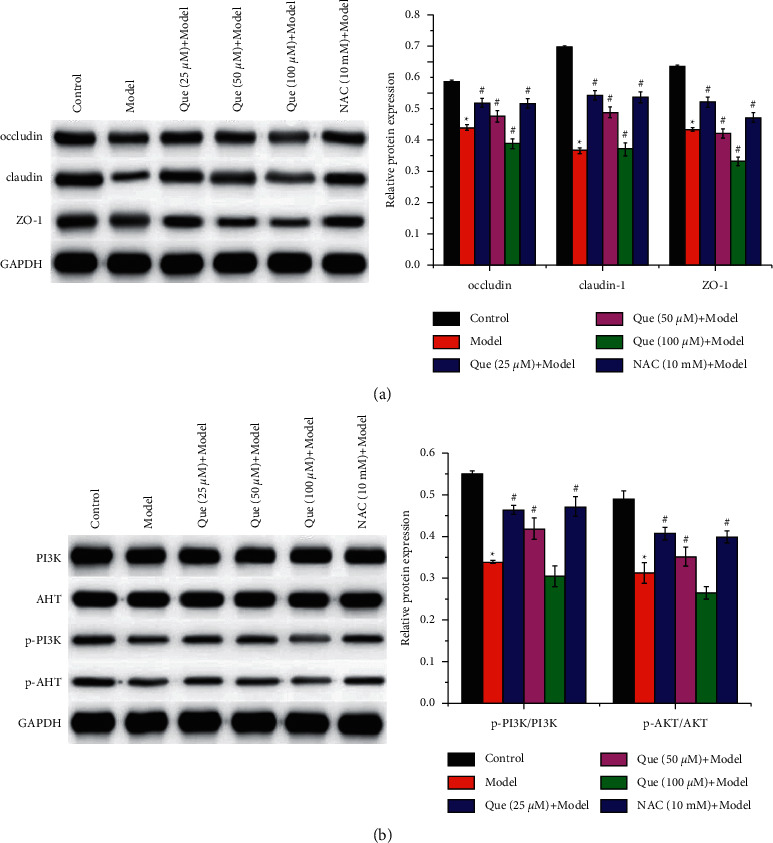
Que improves the cell barrier and activates the PI3K/AKT signaling pathway in H_2_O_2_-induced GES-1 cells. Western blot was performed to detect the (a) tight junction proteins occludin, claudin-1, and ZO-1 and (b) PI3K/AKT signaling pathway-related protein expression in GES-1 cells. Data presets as mean ± SD, n− = 3, ^∗^*P* < 0.05 vs. control group, ^#^*P* < 0.05 vs. model group.

Western blot was used to detect the expression of tight junction proteins, such as occludin, claudin-1, and ZO-1. The results suggested that the decreased expression of occludin, claudin-1, and ZO-1 induced by H_2_O_2_ in GES-1 cells was increased by 25 *μ*M and 50 *μ*M of Que and NAC treatment (Figure 3(a)). Furthermore, the activation of the PI3K/AKT signaling pathway in GES-1 cells was evaluated. The results of western blot showed that H_2_O_2_ suppressed the phosphorylation of PI3K and AKT, whereas they were increased by 25 *μ*M and 50 *μ*M of Que and NAC treatment (Figure 2(b)).

Que improves H2O2-induced oxidative stress damage in GES-1 cells by regulating the PI3K/AKT signaling pathway.

**Figure 4:**
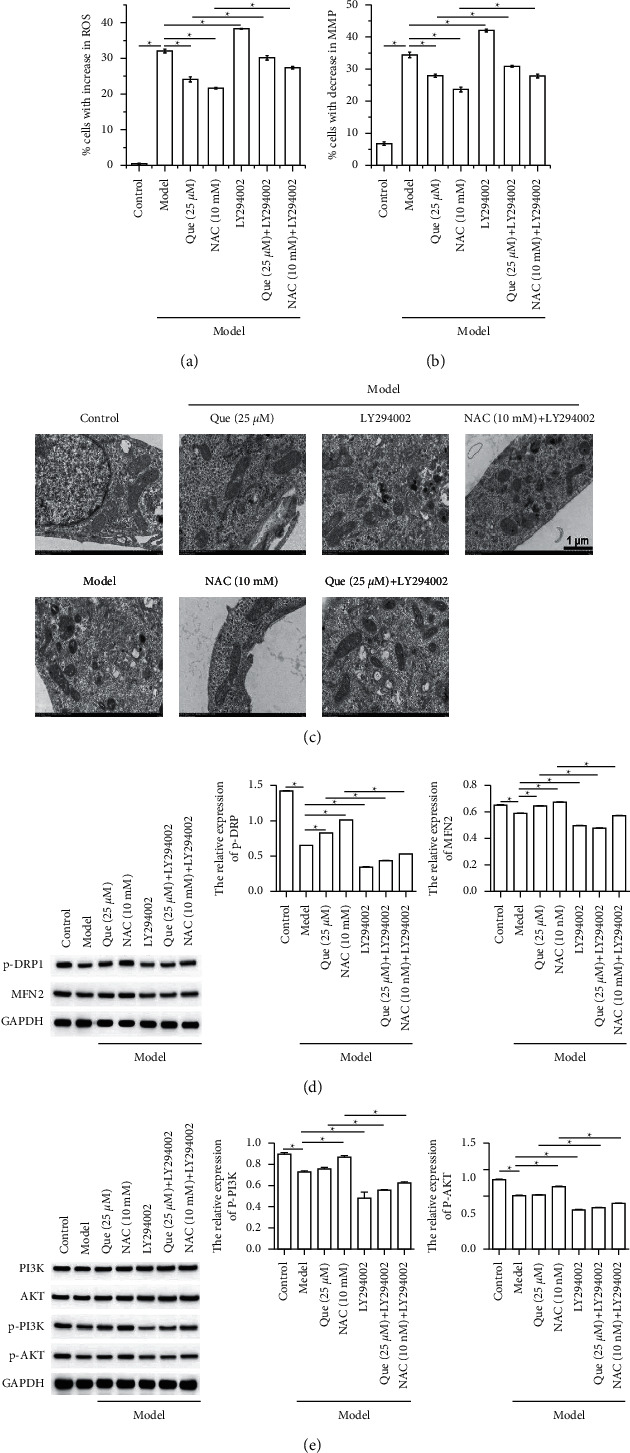
Que improves H_2_O_2_-induced oxidative stress damage in GES-1 cells by regulating the PI3K/AKT signaling pathway. Flow cytometry was performed to evaluate the proportion of (a) cells with increase in ROS and (b) apoptotic cells. (c) The representative ultrastructure of mitochondria in GES-1 cells (12.0 K). Western blot was performed to detect the (d) the phosphorylation of DRP1 and the expression of MFN2 and (e) PI3K/AKT signaling pathway-related protein expression in GES-1 cells. Data presets as mean ± SD, *n* = 3, ^∗^*P* < 0.05.

As shown above, 25 *μ*M and 50 *μ*M of Que treatment activated the PI3K/AKT signaling pathway in H_2_O_2_-induced GES-1 cells. To investigate whether Que alleviated the oxidative stress damage in GES-1 cells by the PI3K/AKT signaling pathway, the GES-1 cells were treated with Que (25 *μ*M) combination with and without LY294002, the PI3K inhibitor. Experiment results showed that compared to the model group, LY294002 further increased the proportion of cells with an increase in ROS generation (Figure 4(a)) and decrease in MMP (Figure 4(b)), exacerbated the mitochondrial structure in GES-1 cells (Figure 4(c)), and decreased the phosphorylation of DRP1 and the expression of MFN2 (Figure 4(d)). In addition, the decrease proportion of cells with increase in ROS generation and decrease in MMP, the alleviation of mitochondrial structure, and the increase expression of p-DPR1 and MFN2 induced by Que were reversed by LY294002 treatment. Moreover, western blot assay showed that compared to the model group, LY294002 treatment decreased the phosphorylation of PI3K and AKT. Compared to Que treated cells, LY294002 combination treatment decreased the phosphorylation of PI3K and AKT.

## 4. Discussion

The present work reported the significant effect of Que on oxidative stress damage in GES-1 cells. High concentration of Que (100 *μ*M) showed cytotoxicity in H_2_O_2_-induced GES-1 cells. However, appropriate concentration of Que (25 and 50 *μ*M) alleviated the oxidative stress damage induced by H_2_O_2_, as demonstrated by the increase of proliferation, decrease of ROS generation, apoptosis, inflammation, and alleviation of mitochondrial function and mucosal barrier. Inflammation is one of the pathological characteristics of FD [23,24]. Turkkan et al. found that *Helicobacter pylori-*induced FD might be related with it evoked oxidative stress and inflammation [25]. D-galacturonic acid suppressed the inflammatory response in rats, thereby alleviating FD symptom [26]. Variety sources contributed to inflammation, including exposure to radiation, toxic chemicals, and allergens, microbial infections, autoimmune diseases, consumption of alcohol, and a high-calorie diet [27,28]. Accumulating research revealed that oxidative stress can result in inflammation, which in turn contributes to several diseases, such as cardiovascular, cancer, diabetes, and gastrointestinal disorders [29,30]. Oxidative stress has been reported to be involved in the activation of various transcription factors including NF-*κ*B, p53, *β*-catenin/Wnt, and Nrf2, which were associated with the expression of inflammatory cytokines, thereby participating in the regulation of inflammation [29,31–33]. Our present work showed that appropriate concentration of Que increased the level of IL-4, called as “prototypic immunoregulatory cytokine” that has anti-inflammatory function [34] and decreased the level of TNF-*α*, which induced inflammation [35], indicating the inhibition effect of Que on inflammation in H_2_O_2_-induced GES-1 cells.

ROS is mainly generated form mitochondria, wherein oxidative stress focused damage. The mitochondrial permeability transition pore structure and function are closely related to the mitochondrial biological function. In general, the opening and closing of the mitochondrial permeability transition pore (MPTP) is under balance condition, which contributes to intracellular calcium ions balance [36]. However, oxidative stress accelerates the opening of MPTP, resulting in the aggregation of protein macromolecules, thereby leading to the matrix osmotic swelling and dysfunction of mitochondria [37,38]. It has been identified that oxidative stress enhanced the opening of MPTP partially due to its regulated effect on Bcl-2 family protein [39,40]. The sustainable opening of MPTP leaded to the decrease of MMP and the cytochrome C release into the the cytosol [41], thereby promoting caspase 9 formation and the intracellular apoptosis-related proteins (such as caspase 3) proteolytic cleavage, which induced apoptosis [42–44]. Except as described above, the integrity of mitochondrial physiological function is also determined by the morphological changes of mitochondria, which is mainly regulated by mitochondrial fission and fusion genes. Evidence verified that oxidative stress is involved in changing the balance of mitochondria fission and fusion through modulating DRP1, a mitochondrial fission-associated protein, and MFN2, a mitochondrial fusion-associated protein, in turn inducing energy metabolism disorder-mediated mitochondrial apoptosis [45–47]. Our current study indicated that compared to Model group, Que decreased the proportion of cells with an increase in ROS generation and decrease in MMP, alleviated the swollen malformation of ridges in mitochondria, and increased the phosphorylation of DRP1 and the expression of MFN2 and Bcl-2, and decreased the expression of Bax and cleaved caspase 3, indicating the improvement effect of Que on mitochondrial dysfunction induced by oxidative stress response. In addition, this work reported that Que repairs the cellular barrier by regulating tight junction proteins occludin, claudin-1, and ZO-1 [48, 49].

PI3K/AKT signaling pathway is an important intracellular functional regulatory pathway that involves in a variety of biological process including proliferation, apoptosis, migration, invasion, and oxidative stress [49,50]. These biological functions enable it to participate in the regulation of a variety of diseases, such as cancer [10], diabetes [51], neurodegenerative diseases [52], and gastrointestinal disorders [53]. This work showed that appropriate concentration of Que increased the activation of phosphorylation of PI3K and AKT decreased by H_2_O_2_. PI3K/AKT signaling inhibition suppressed the alleviation effect on Que in H_2_O_2_-induced GES-1 cells. These indicated that Que alleviates oxidative stress damage in GES-1 cells might be mediated by the PI3K/AKT signaling pathway.

In conclusion, our current study gave evidence which suggested that Que alleviates oxidative stress damage in GES-1 cells by improving mitochondrial function, cell barrier and suppresses inflammation. The underlying molecular mechanism might be associated with its regulation effect on the PI3K/AKT signaling pathway. The limitation of this study is the absence of in vivo experiment, which will be designed in the follow-up study to further strength our current conclusion. The present work identified Que as a potential candidate for FD therapy.

## Data Availability

The data used to support the findings of this study are included within the article.
